# Thin-Walled Cylindrical Shell Storage Tank under Blast Impacts: Finite Element Analysis

**DOI:** 10.3390/ma14227100

**Published:** 2021-11-22

**Authors:** Ahmad Mahamad Al-Yacouby, Lo Jia Hao, M. S. Liew, R. M. Chandima Ratnayake, Samindi M. K. Samarakoon

**Affiliations:** 1 Civil and Environmental Engineering Department, Universiti Teknologi PETRONAS, Seri Iskandar 32610, Perak, Malaysia; lo.jia_24018@utp.edu.my (L.J.H.); shahir_liew@utp.edu.my (M.S.L.); 2 Department of Mechanical and Structural Engineering and Materials Science, University of Stavanger, 4021 Stavanger, Norway; chandima.ratnayake@uis.no (R.M.C.R.); samindi.samarakoon@uis.no (S.M.K.S.)

**Keywords:** thin-walled cylindrical shell, storage tank, blast impacts

## Abstract

Thin-walled cylindrical shell storage tanks are pressure vessels in which the walls of the vessel have a thickness that is much smaller than the overall size of the vessel. These types of structures have global applications in various industries, including oil refineries and petrochemical plants. However, these storage tanks are vulnerable to fire and explosions. Therefore, a parametric study using numerical simulation was carried out, considering the internal liquid level, wall thickness, material yield strength, constraint conditions, and blast intensity, with a diameter of 100 m and height of 22.5 m under different blast loads using the finite element analysis method. The thickness of the tank wall is varied as 10 mm, 20 mm, 30 mm, and 40 mm, while the fill level of internal fluid is varied as 25, 50, 75, and 100%. The blast simulation was conducted using LS-DYNA software. The numerical results are then compared with analytical results. The effects of blast intensity, standoff distance, wall thickness, and fill level of internal fluid on the structural behaviour of the storage tank were investigated and discussed.

## 1. Introduction

Thin-walled cylindrical shell storage tanks are found in many commercial and industrial applications. These tanks are important facilities in the oil and gas and petrochemical industries, for they store large volumes of flammable, explosive, toxic, and harmful materials. With the rapid development of the global economy and the strategic demand and production demand of energy, the volume and quantity of storage tanks are increasing, and consequently, the scale of tank farms is expanding, showing characteristics of large-scale integration and coexistence of multiple tanks [1,2,3]. These storage tanks are subjected to internal pressure, which subjects the tanks to a uniform loading, considering that the tanks have an inner-radius-to-wall-thickness ratio of 10 or more [4]. In the last few decades, a number of major industrial accidents have occurred around the world [5,6]. Blast loading to the exterior of a cylindrical shell pressure vessels imposes severe consequences [7]. Despite the high risk of explosions in the oil and gas industry, little related research can be found in the literature, especially on the effect of tank dimensions and standoff distance from blast on the behaviours of thin-walled cylindrical shells.

The explosions happen when the stored hydrocarbon-air mixtures present above the liquid state are exposed to adequate amounts of energy [8]. Because of these devastating explosions, a few researchers have carried out studies and reviews to investigate the main causes of storage tank accidents. According to Chang and Lin [9], among 242 tank accidents from 1960–2003, 74% happened in petroleum refineries, oil terminals, and storage. In total, 33% of the cases were caused by lightning, and another 30% were caused by human error. A review of the literature shows that hydrocarbon explosions caused 70% of the accidents in offshore installations as well. Accidents are prone to happening during maintenance, repair, and loading or unloading. Other research found that 27% of the accidents were due to human error, including operations and maintenance [10]. Thus, understanding how these types of thin-walled structures respond to relevant loading conditions is important for the design of safe and economical liquid-containment shell storage tanks [2]. However, hardly any progress has been made in understanding the dynamic response of liquid storage tanks under blast loading [11]. A review of the literature shows that several researchers [6,12,13,14] have investigated the influence of blast intensity, tank fill conditions, and tank bottom constraints on tanks’ failure mode, resultant displacement and deformation, structural energy, circumferential strain, and longitudinal strain. Moreover, small-scale model experiments have been mostly conducted to study the response of thin-wall cylindrical tanks subjected to blast load [3,6], and the findings have been verified using numerical simulations. However, in order to study the dynamic response of large thin-walled cylindrical tanks, the applicability of numerical simulations has been widely discussed. Hence, recent advances in numerical simulation have provided a powerful tool to examine increasingly complex problems involving explosive blast loading [15,16,17].

Mittel et al. [11] carried out a parametric study by varying the dimensions of the small thin-walled cylindrical tank height-to-radius ratio (0.5 to 2.6), percentage liquid filling (50% to 100%), thickness of tank wall (1 mm to 10 mm), and scaled distance of the explosive material (0.5 to 2.5 m/kg^1/3^). The numerical simulations of the study show that there is a significant influence of the thickness of the tank wall and the height-to-radius ratio on plastic yielding. The study demonstrated the importance of studying the influence of the dimensions of the tank on the overall performance of the tank. In addition, there are very few studies in the literature on the use of numerical simulations to study the dynamic response of large thin-walled cylindrical tanks where the diameter of the tank is greater than the height of the tank. For example, Lu et al. [6] have discussed the applicability of numerical simulations for a thin-walled cylindrical tank which has diameter of 100 m and height of 22.5 m. Authors [6] have studied the structural response by varying the internal liquid level, constraint conditions, and blast intensity while maintaining constant tank wall thickness, height, and diameter. For more comprehensive reviews and the latest studies on thin-walled pressure vessels, the reader can refer to [18,19,20]. However, the literature does not report clear and exact statements regarding the influence of a tank’s dimensions and the distance from the blast on the overall performance of a thin-walled cylindrical shell storage tank under blast loading. Thus, the aim of the present investigation is to study the behaviour of a thin-walled cylindrical shell storage tank with a diameter of 100 m and a height of 22.5 m under different blast loads using the finite element analysis method. The thickness of the tank wall is varied as 10 mm, 20 mm, 30 mm, and 40 mm, while the fill level of the internal fluid is varied as 25, 50, 75, and 100%. The outline of the remaining sections is: Section 2 provides the theoretical background of the analytical approach; Section 3 introduces the materials and methods adopted in this study; Section 4 presents the results and discussion; and the conclusion is presented in Section 5.

## 2. Theoretical Background

Cylindrical pressure vessels are commonly used in the oil and gas industry to carry both liquid and gases under pressure. When the vessels are exposed to this pressure, the materials comprising the vessels will be subjected to longitudinal and hoop stress. The important assumptions to be considered in deriving the equations of hoop and longitudinal stress are:

- Plane sections remain plane.
- Radius-to-thickness ratio greater than or equal to 10 with uniform and constant wall thickness.
- Linear elastic, isotropic, and homogeneous material.
- Uniform stress distribution throughout the wall thickness.
- Negligible fluid weight.

The circumferential stress in a thin-walled cylindrical shell storage tank can be determined using the following Equation (1):

(1) $$\sigma_{h}=\frac{Pr}{t}$$

where *P* is internal pressure, *r* is mean radius of the cylinder, and *t* is wall thickness.

The longitudinal stress in a pressurised thin-walled cylindrical shell storage tank can be expressed using the following Equation (2):

(2) $$\sigma_{L}=\frac{Pr}{2t}$$

The equivalent tensile stress known as Von Mises stress can be computed using Equation (3). This stress can be used to estimate the yielding of materials under complex loadings. The Von Mises failure criterion is one of the most-used failure criteria for ductile materials such as structural steel.

(3) $$\sigma_{v}=\sqrt{\sigma_{h}{}^{2}+\sigma_{L}{}^{2}-\sigma_{h}\sigma_{L}}\;$$

where *δ_h_* and *δ_L_* are the hoop stress and the longitudinal stress, respectively.

## 3. Materials and Methods

The research methodology adopted in this study is discussed in the following sections.

### 3.1. Storage Tank Geometry

The main parameters influencing the structural behaviour of a thin-walled cylindrical storage tank are namely the height of the tank, the outer diameter, the wall thickness, and the yield strength of material. The material of the tank has a density of 7850 kg/m^3^ and a Poisson ratio of 0.28. The additional modelling parameters for the thin-walled storage tank, provided by an oil and gas company operating in Malaysia, are presented in Table 1. The model with all the different parameters was subjected to internal pressure resulting from hydrocarbon product with a density of 800 kg/m^3^. In this study, a storage tank with higher capacities was selected for modelling, as there is a global trend in storage capacities greater than 20 × 10^4^ m^3^ and diameters greater than 100 m in the oil and gas industry [21,22].

### 3.2. Numerical Modelling

**Figure 1:**
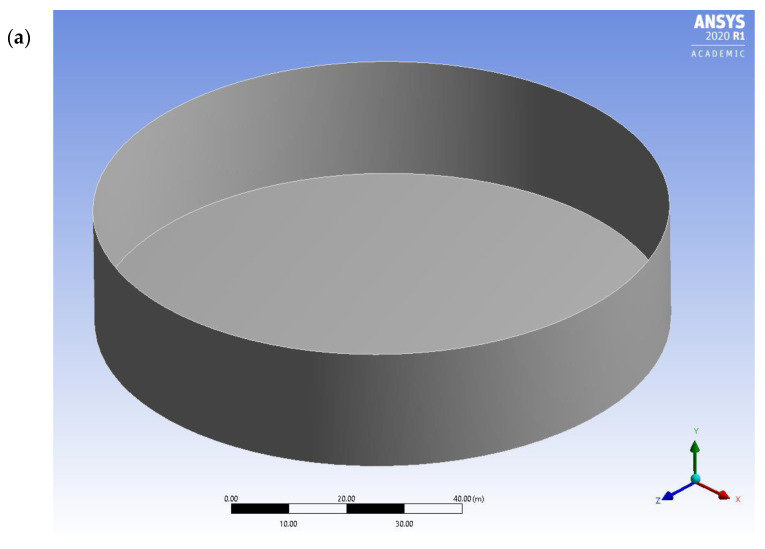

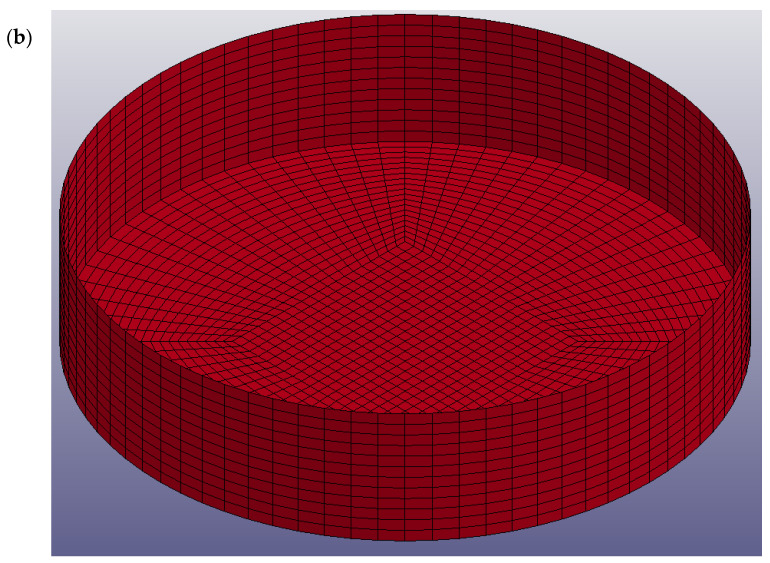
(**a**) Proposed model of thin-walled storage tank. (**b**) Meshing details.

The numerical simulation for the selected parameters consists of three stages, namely pre-processing, simulation, and post-processing. During the pre-processing stage, ANSYS Workbench 2019 R19.1 software from ANSYS, Inc. (Washington County, PA, USA) was used for developing the geometry, assigning the material properties, loading, and finalising the boundary conditions of the model. The definitions of all properties and design parameters must be precise and accurate to ensure the case study is correctly simulated in the software. The model geometry was then meshed into smaller elements connected by nodes to perform the finite element analysis (FEA). Generally, the sensitivity analysis shows that a smaller mesh will produce results with higher accuracy, but consequently, it will lead to higher processing time and cost. Hence, as the wall thicknesses of the tank are much smaller than the other geometric parameters, the tank was meshed using shell elements [17]. The model is a simplified representation of the storage tank, with the tank wall and base plate modelled as SHELL181 elements to increase the analysis accuracy and the efficiency of the simulation. The SHELL181 element has four nodes with three translational and three rotational degrees of freedom at each node, and linear interpolation is used within the element [23]. In explicit dynamics, by default, coarse relevance centre, high smoothing, and slow transition were selected. The elements should have a uniform size to ensure the meshing quality. To ensure accuracy of the results, convergence and mesh independence analysis was conducted first. The optimum set of element sizes was adopted when the maximum displacement of the tank had insignificant influence when coarser element sizes were tested in the numerical model. As a result, the maximum element size in the numerical simulation was 13 mm × 20 mm, considering a maximum number of 128 K-nodes/elements. The thicknesses of the shell elements were defined as of 10 mm, 20 mm, 30 mm, and 40 mm, respectively, based on the specifications in API 650 [22]. Once the geometry, meshing, load assignment, and boundary conditions were found satisfactory, the analysis was done to solve the model using the governing equations. The outputs such as forces, stresses, and deformations of the model were generated based on different loading cases. The post-processing is the stage where the output from the simulation is reviewed. The results in different forms, such as contour, graphs, and tables, will provide the necessary information on the model properties. In this study, more emphasis was placed on the resultant deformation and Von Mises stress of the cylindrical storage tank. The proposed tank and meshing details are presented in Figure 1.

### 3.3. Blast Modelling

Using the parametric study presented in the previous section, the optimum thin-walled pressure vessel design was determined. The optimum model was then subjected to blast loading with different intensities from different distances. Ansys LS-DYNA 2019 R19.1 software from ANSYS, Inc. was used in the blast simulation, as the high impact velocity of the blast causes higher stress, strain rate, local deformation, and pressure in a short duration. The detonation was modelled using the Lagrange approach, as this approach normally requires a lesser mesh count as compared to the Eulerian approach and has a much shorter computational time. The material assigned will be embedded within the mesh and move along with the mesh. The blast loading in the finite element model of this study is modelled using Load Blast Enhanced (LBE), which is a fully Lagrangian approach used for air blast loads from conventional explosives. The air blast pressure was simulated empirically based on experimental data from The conventional weapons effects blast loading model (ConWep), converted into polynomials through classical scaling laws as reported in [24], and then applied to the nodes of a Lagrangian structure. The cylindrical steel tank was modelled using a material of Mat Piecewise Linear Plasticity keywords, where the strain rate parameters *C* and *P* of Cowper – Symonds relation are set to 40 and 5, respectively, to take the plastic deformation and the strain rates into consideration. The termination time was set to 0.25 s with a timestep of 0.0005 s to capture the effect of blast load [25]. The waveform of the blast is described using the Friedlander waveform, where the pressure of the blast wave is described as a function of time as presented in Equation (4) [26].

(4) $$P\left(t\right)=P_{s}e^{-\frac{t}{t^{*}}}\left(1-\frac{t}{t^{*}}\right)$$

where *P_s_* is the overpressure (pressure above ambient pressure) and t* is the duration of the positive phase, when the pressure is higher than the ambient pressure.

The flammable gas volumes adopted in this study were 15,600 m^3^ and 4000 m^3^. These volumes had blast intensities of 1500 kg and 380 kg of Trinitrotoluene (TNT), respectively. The proposed standoff distances of the blast from the storage tank were 12.5 m and 25 m, respectively, at a three-meter height, assuming an explosion from gas leakage, and the structural responses of the tank under 100 and 0% fill were investigated. The blast scaling law or cube root scaling law, proposed by Hopkinson, was used to represent the relationship between the weight of the explosive charge and the standoff distance from the explosive charge centre. The usage of the scaling law shown in Equation (5) is to predict the effects of large-scale explosions by conducting experiments on an adequately scaled specimen.

(5) $$Z=\frac{R}{W^{\frac{1}{3}}}$$

where *R* is the distance from the explosive charge centre and *W* is the weight of the explosive charge.

The blast pressure generated from LS-DYNA was validated using analytical methods from the commonly used empirical equations, such as the Brode equation [27]. The peak overpressure calculated from the analytical method was compared with the blast pressure obtained from simulations as a check. The Brode equations presented in Equations (6) and (7) are based on differential equation formulations for pressure more than 10 bars and pressure ranging from 0.1 to 10 bars, respectively.

(6) $$P_{so}=\frac{6.7}{Z^{3}}+1\;for\;P_{so}>10\;bar$$

(7) $$P_{so}=\frac{0.975}{Z}+\frac{1.455}{Z^{2}}+\frac{5.85}{Z^{3}}-0.019\;for\;0.1<P_{so}<10\;bar$$

where *Z* is the scaled distance in m/kg^1/3^.

## 4. Results and Discussion

This section presents the results obtained through the numerical simulation and the validation of the results using the analytical approach suggested in [27,28].

### 4.1. Parametric Study of a Thin-Walled Cylindrical Shell Storage Tank

In this section, the numerical modelling of the storage tank, developed based on the different specifications defined in Section 3, is presented.

#### 4.1.1. Total Deformation of Different Wall Thickness versus Fill Level

**Figure 2:**
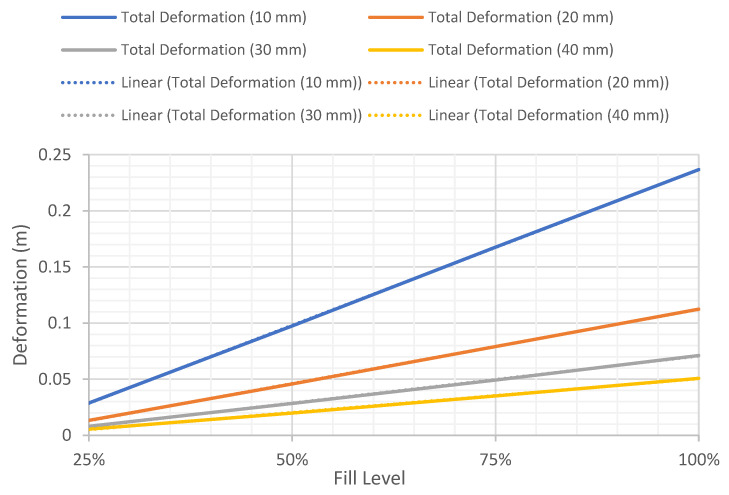
Variation of maximum total deformation of different wall thicknesses versus fill level.

**Figure 3:**
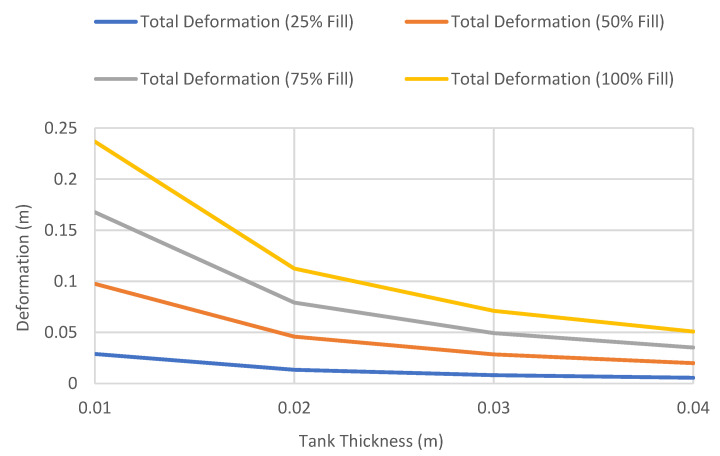
Variation of maximum total deformation of different fill levels versus tank thickness.

**Figure 4:**
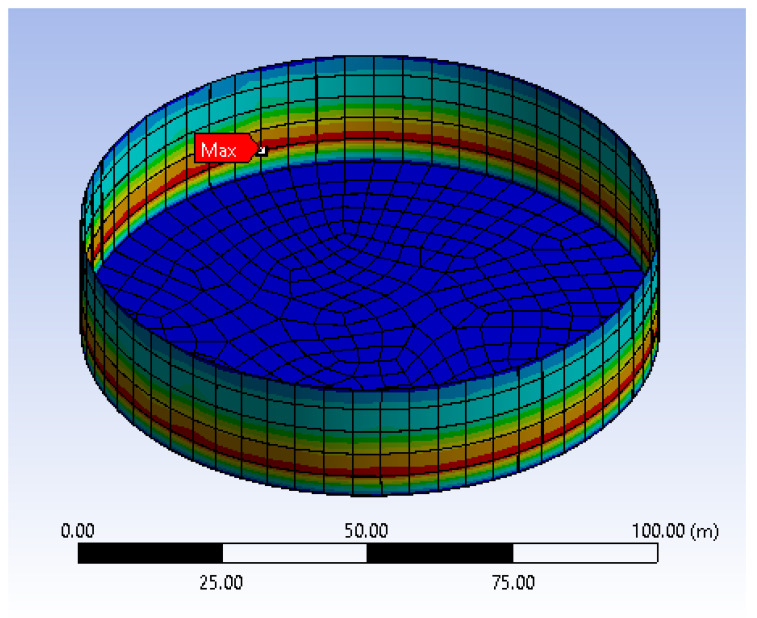
Location of maximum deformation of the tank.

In this section, static analysis was used to determine the relationship between the wall thickness and internal fill levels on the total deformation of the tanks. Figure 2 shows the comparison of the maximum total deformation of different wall thicknesses versus fill level. The thicknesses of the shell elements were defined as 10 mm, 20 mm, 30 mm, and 40 mm, respectively, based on the specifications in API 650. The inner face of the wall was subjected to hydrostatic pressure of 25, 50, 75, and 100% fill level based on a fluid density of 800 kg/m^3^ and a kinetic viscosity of 1.9 mm^2^/s. Based on these defined conditions, a parametric study was conducted to investigate the effect of shell thickness and fill level on the maximum deformation and Von Mises stress of the tank. The result of the analysis shows that the total deformation on the tank increases with increasing the wall thickness. The maximum wall deformation was determined as 0.24 m, corresponding to 100% fill level. Similarly, the effects of wall thickness on the total deformation were also investigated. Figure 3 depicts the maximum total deformation for different fill levels as a function of tank thickness. The graph shows that the total deformation of the tank decreases when increasing the wall thickness, with the maximum deformation recorded as 0.24, corresponding to wall thickness of 0.01 m at 100% fill level. The maximum displacements of the tank as a function of wall thickness, presented in Figure 2, are in good agreement with the findings reported by Jiang et al. [3]. Because of the hydrostatic force incurred by the stored hydrocarbon products and the assigned support at the bottom plate, the storage tank will experience maximum deformation at the lower part of the tank, which is 5 m from the bottom plate as shown in Figure 4. The deformation and fill level were found to have a linear relationship in every shell thickness defined. The thinner the wall of the tank, the steeper the gradient of the graph, indicating that the deformation of tanks with a lower thickness is more sensitive to the changes of fill level. It can be seen that the tank will deform the most when it is 100% filled as compared to other partial fill levels. As expected, the deformation increases with higher fill levels and decreases with thicker tank walls. However, the deformations are very minimal and do not impact the serviceability and structural integrity of the tank. Thus, more focus is given to the equivalent stress known as the Von Mises stress and its variation, with respect to the yielding stress of the storage tank, as discussed in the following section.

#### 4.1.2. Von Mises Stress versus Fill Level of Different Tank Thicknesses

**Figure 5:**
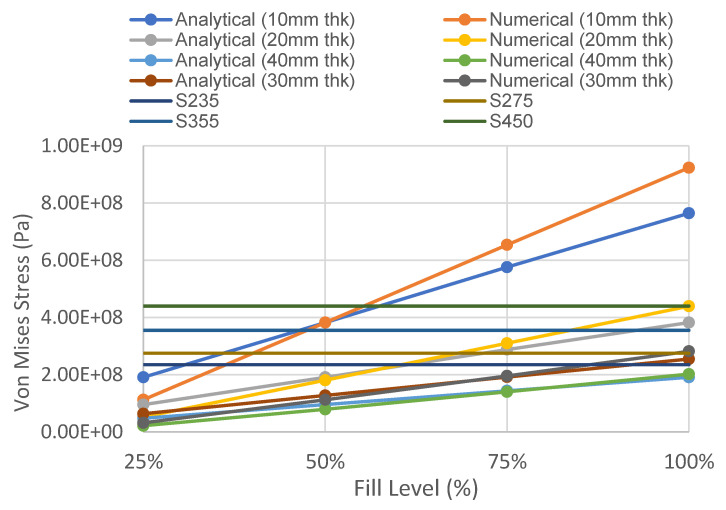
Variation of Von Mises stress versus fill level of different tank thicknesses.

**Figure 6:**
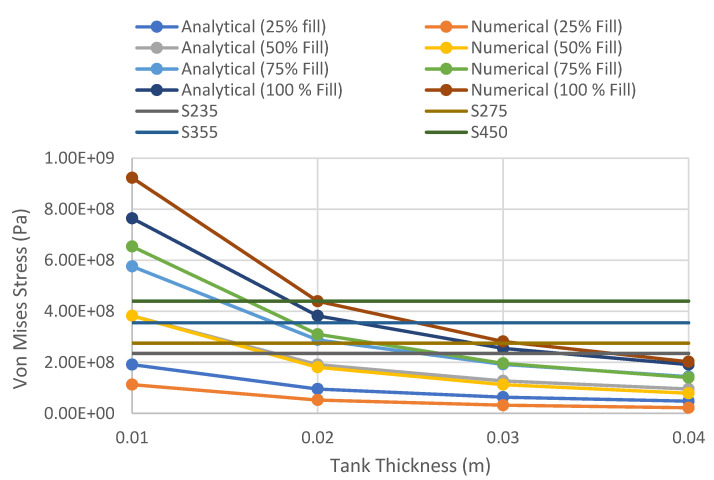
Variation of Von Mises stress versus tank thickness of different fill levels.

The variations of Von Mises stress versus the fill level of different tank thicknesses and various wall thicknesses are presented in Figure 5 and Figure 6, respectively. The comparison of the Von Mises stress between an analytical approach and numerical modelling is focused on a storage tank with 100% fill level, as the usage of simplified equations assumed a uniform stress distribution throughout the surface of the tank. Based on the comparison between the analytical method and the numerical results, it was found that the results are generally in good agreement with each other, having similar values and having almost the same trend for the different tank thicknesses. The highest percentage difference between the numerical results and the analytical value of equivalent stress was observed to be 17%, when the shell thickness is 10 mm and the fill level is 100%. As the shell thickness of the tank increases, the difference between the numerical and analytical values reduces. The efficiency of each steel grade is also evaluated based on the Von Mises stress at 100% fill level to ensure the storage tank will not yield under normal usage. For a large-scale storage tank, the yield strengths of S235 steel and S275 steel are easily exceeded with a tank thickness of less than 20 mm. This is in accordance with API 650′s specification for EN 10025 S 355 J0, J2, and K2 structural steel plates, with the minimum yield strength being 355 MPa. The S450 structural steel will permit the usage of 20-mm-, 30-mm-, and 40-mm-thick plates, while S355 permits 30 mm and 40 mm plate thicknesses. By using the higher steel grade of S450, adequate structural capacity will be achieved even with 20-mm wall thickness. The response of the finite element model with the 20-mm tank wall and base plate made of S450 structural steel subjected to blast load will be discussed in the following section.

### 4.2. Validation of Blast Modelling Using Analytical Equations

**Figure 7:**
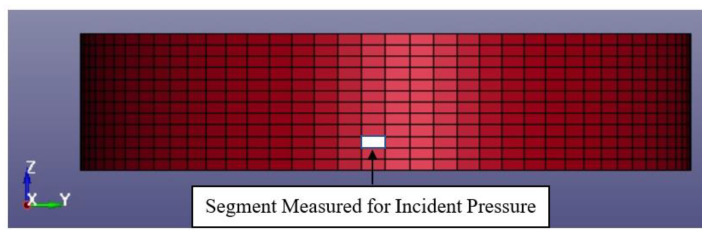
Typical segment of the tank where incident pressure is measured.

Blast simulations of an S450 storage tank with 20-mm wall thickness were carried out for each case defined in Section 3 with a run of 0.25 s and with a time step of 0.005 s. Time history of the incident pressure is measured from the selected tank segment facing the blast, centred 4.69 m from the bottom plate. The incident pressures of each blast case were then compared with the value obtained from the analytical method for validation purposes. A typical segment of a tank with the meshing details used is shown in Figure 7.

**Figure 8:**
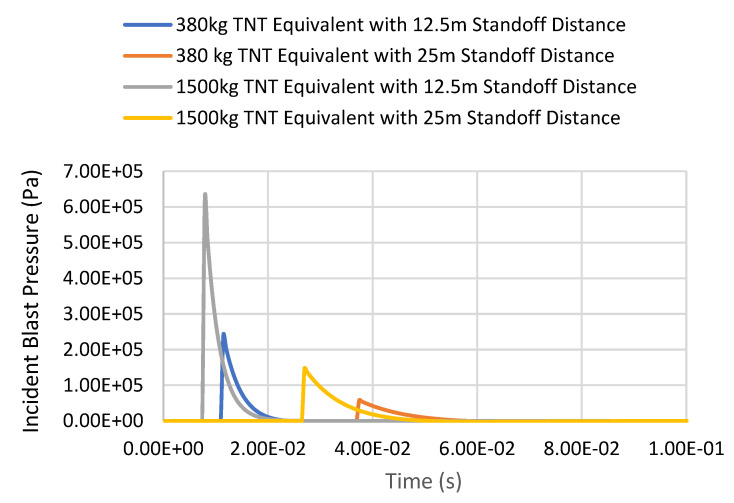
Incident blast pressure versus time graph of all blast cases.

Figure 8 shows the incident blast pressure versus time graph of all blast cases. The peak pressure time history for each blast case showed a similar trend to the Friedlander waveform, with the peak pressure at arrival time followed by exponential decay. The peak blast pressure values from numerical modelling and the Brode equation were then compared to find the discrepancies between the analytical approach and numerical approach. The influence of distance on the blast positive pressure phase presented in the figure is in good agreement with [28].

Table 2 summarises the scaled distance, arrival time, and peak pressures determined through the numerical simulations and the empirical equations. From the table, it was found that the scaled distance and arrival time obtained from the model have an inversely proportional relationship; as the scaled distance increases, the arrival time will decrease. The comparison of the results between the blast pressure from the numerical and the analytical approach is presented in Table 3. Practically, the formulas often used to calculate overpressure are the Henrych formula [29], the Brode formula [30] and the Friedlander formula [31], and in this paper, the blast pressure generated from the LS-DYNA simulation was validated using the Brode equation. Generally, the comparison of the results shows that the percentage of difference between the numerical modelling and the analytical approach varies from 5.6 to 12%. The maximum percentage difference of 12% is found when the blast intensity is 1500 kg TNT equivalent with a standoff distance of 25 m, as depicted in Table 3. It is apparent that the results obtained from the numerical modelling are in good agreement with the analytical approach.

### 4.3. Tank Deformation after the Blast Impact

**Figure 9:**
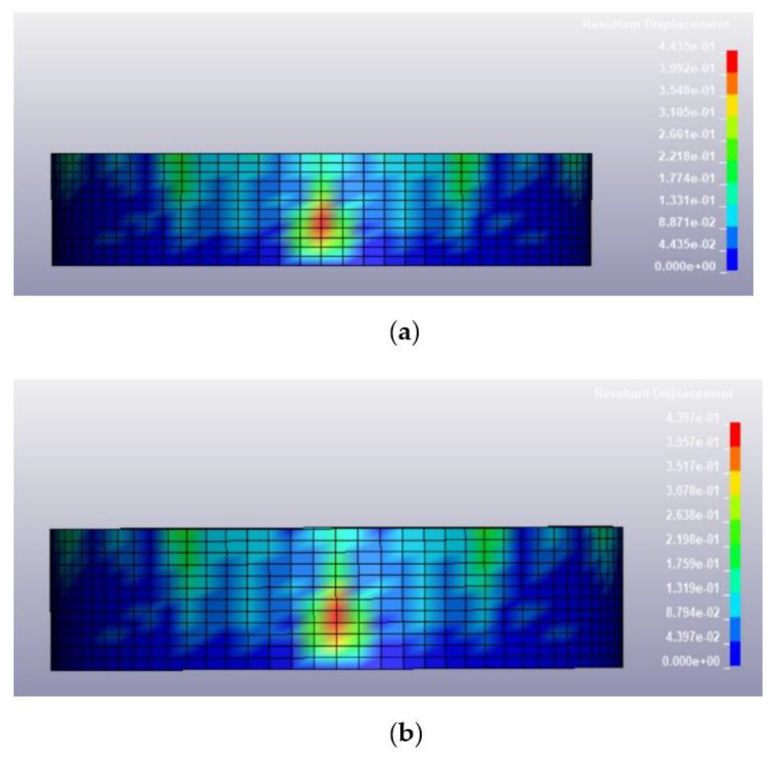
Deformed shape of the tank under blast intensity of 1500 kg TNT equivalent mass 12.5 m away, (**a**) 100% fill level and (**b**) 0% fill level.

**Figure 10:**
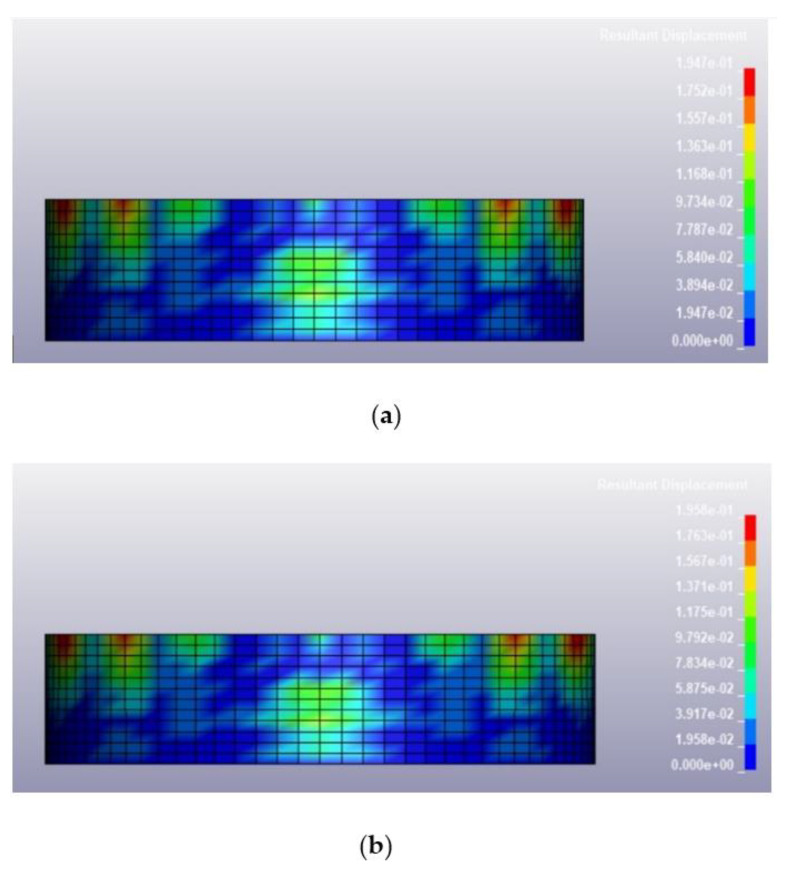
Deformed shape of the tank under blast intensity of 1500 kg TNT equivalent mass 25 m away, (**a**) 100% fill level and (**b**) 0% fill level.

**Figure 11:**
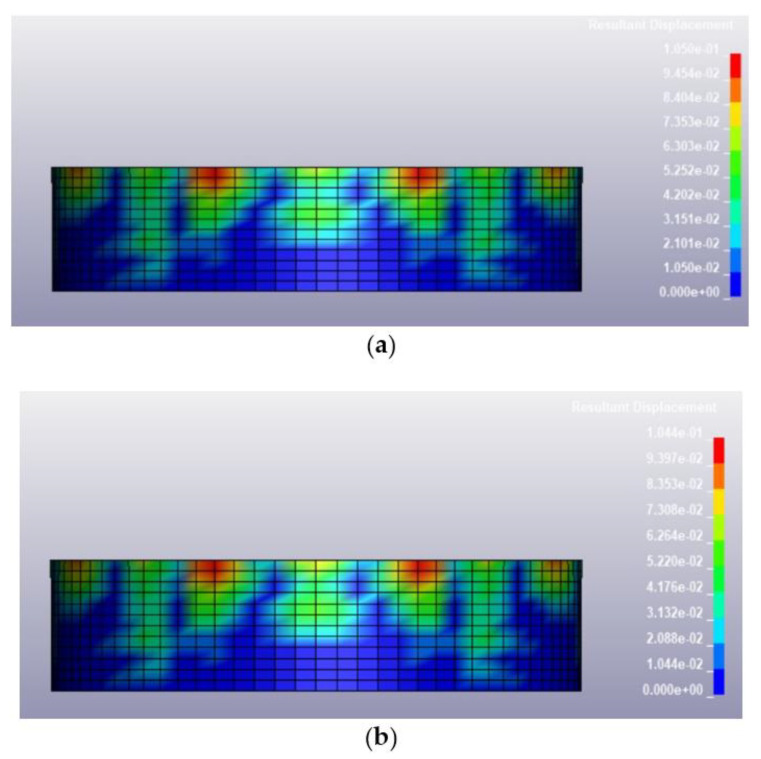
Deformed shape of the tank under blast intensity of 380 kg TNT equivalent mass 12.5 m away, (**a**) 100% fill level and (**b**) 0% fill level.

**Figure 12:**
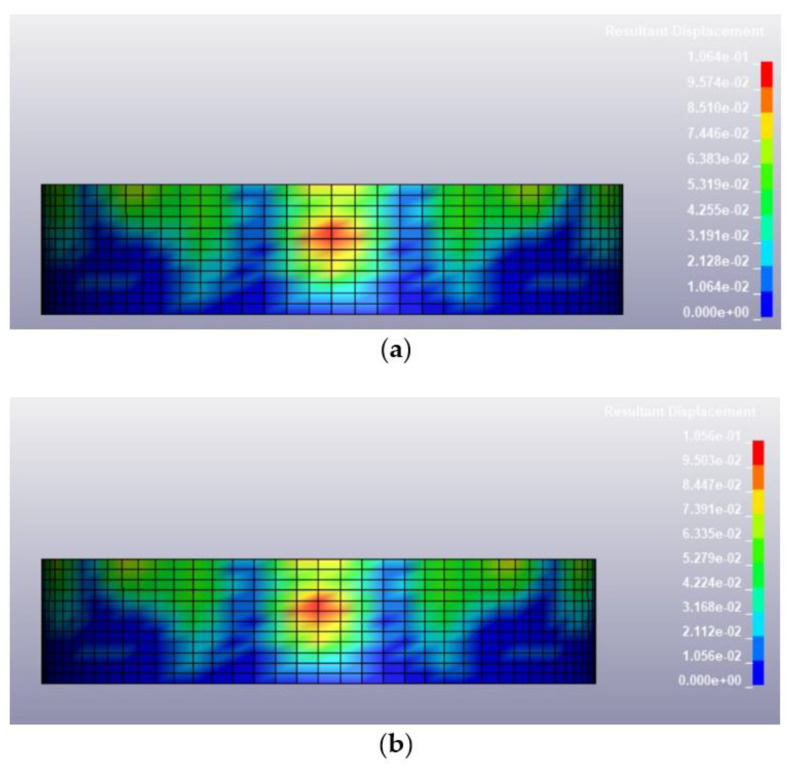
Deformed shape of the tank under blast intensity of 380 kg TNT equivalent mass 25 m away, (**a**) 100% fill level and (**b**) 0% fill level.

Once the validation of the blast pressure of the model was conducted successfully, the finite element model described in the previous section was loaded under various blast conditions, including a blast intensity of 1500 kg TNT equivalent at blast distances of 12.5 m and 25 m for fill levels of 100 and 0%, respectively. The deformed shapes of the tanks at 0.25 s after the blast for different blast conditions are illustrated in Figure 9, Figure 10, Figure 11 and Figure 12. Generally, the storage tank showed almost the same pattern of displacement with different fill levels despite the existing difference in the magnitudes. In Figure 9, the deformed shapes of the storage tank at both fill levels subjected to 1500 kg TNT equivalent blast load from a distance of 12.5 m are found to have the maximum concave deformation at the lower part of the tank. However, in Figure 10, when the distance from the blast was increased to 25 m, the maximum deformation was found to be at the upper part of the tanks. For a blast load of 380 kg TNT equivalent, the maximum deformations when the blast distance was 12.5 m were found to be located at the upper side of the tank as shown in Figure 11, while for the loading case with a blast distance of 25 m, the tank deformation was at its middle panel as shown in Figure 12, which faced the explosion source. The difference in the location of maximum deformation is because of the different propagation of blast waves cause by the varying blast distance and blast intensity.

Based on the summary of tank displacement at 0.25 s, which is the termination time of the analysis presented in Table 4, the tank structure will experience greater displacement at a higher blast intensity and shorter distance from the blast. The maximum displacement observed on the tank was 443.5 mm, corresponding to a blast loading of 1500 kg TNT equivalent and a standoff distance of 12.5 m. The variation of fill level didn’t affect the maximum displacement much, as the highest percentage difference of deformation between 100% fill and 0% fill is merely 0.85%. This is because the density of 800 kg/m^3^ is relatively low and does not affect the displacement significantly. The summary of the maximum tank displacements based on simulations is presented in Table 4. The deformation process and stress and strain distribution of the cylindrical shell are similar to that in the study of Paul et al. [32] and Jian et al. [3], in which the resistance of a thin-walled cylindrical shell with fixed ends is attributed to the axial tension and dynamic pulse buckling of the loaded surface.

### 4.4. Tank Resultant Displacement under Blast Impacts

**Figure 13:**
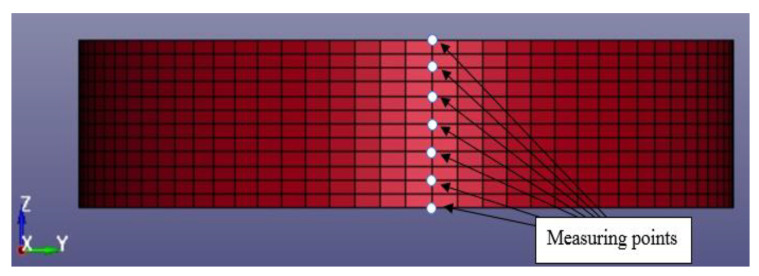
Location of measuring points.

Besides studying the tank deformation after the blast, the resultant displacement of the tank summated from different displacement vectors throughout the explosion was also measured and analysed. The resultant displacement throughout the whole analysis of 0.25 s is measured at seven measuring points, namely S1–S7, at the tank surface facing the blast loading. The locations of the measuring points are shown in Figure 13, while the corresponding coordinates of each reference point are shown in Table 5.

**Figure 14:**
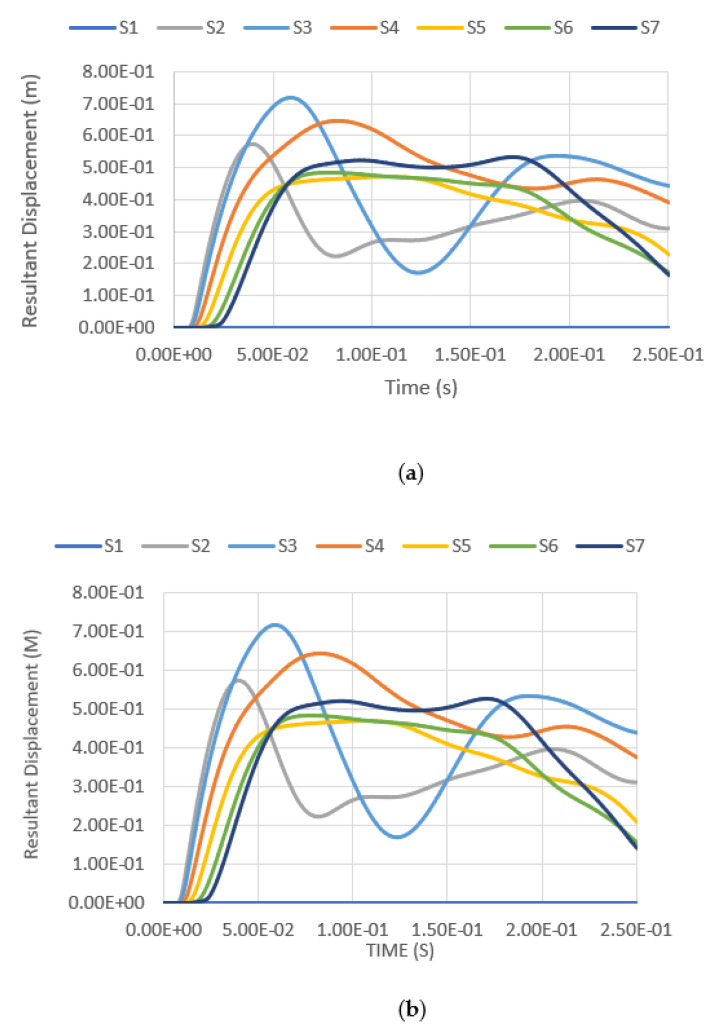
Resultant displacement versus time under blast intensity of 1500 kg TNT equivalent mass 12.5 m away, (**a**) 100% fill and (**b**) 0% fill.

**Figure 15:**
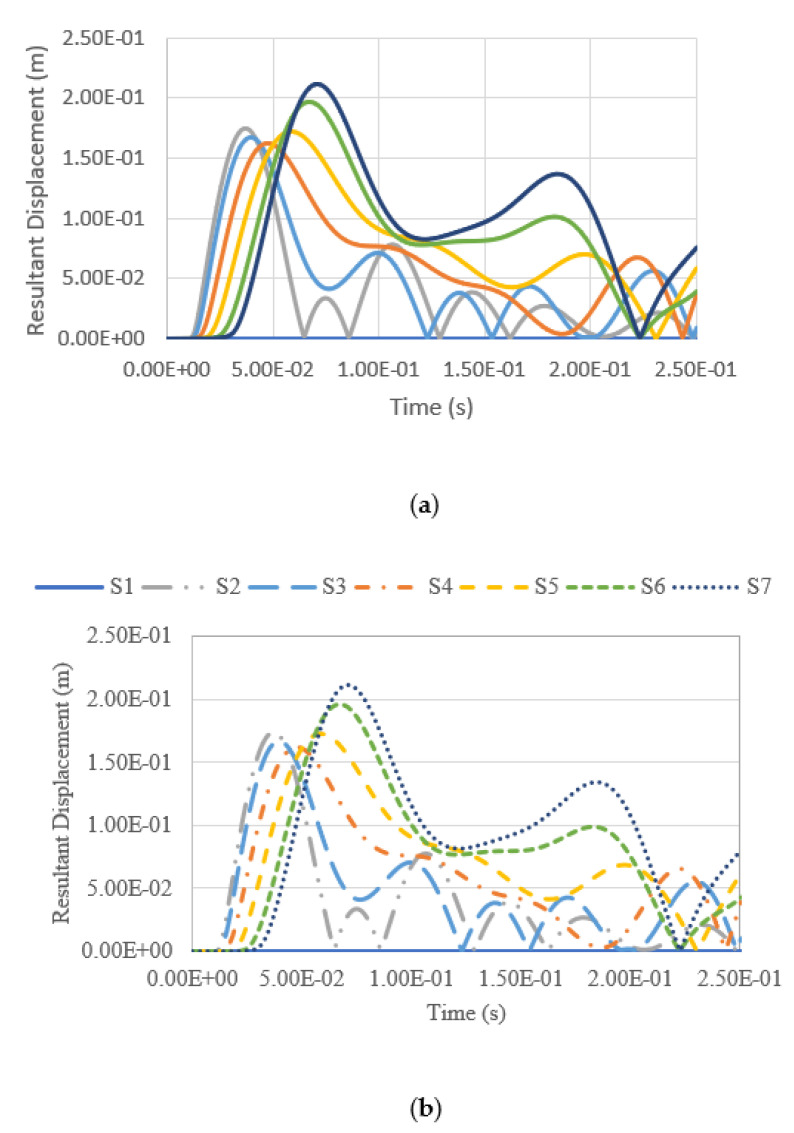
Resultant displacement versus time under blast intensity of 1500 kg TNT equivalent mass 25 m away, (**a**) 100% fill and (**b**) 0% fill.

**Figure 16:**
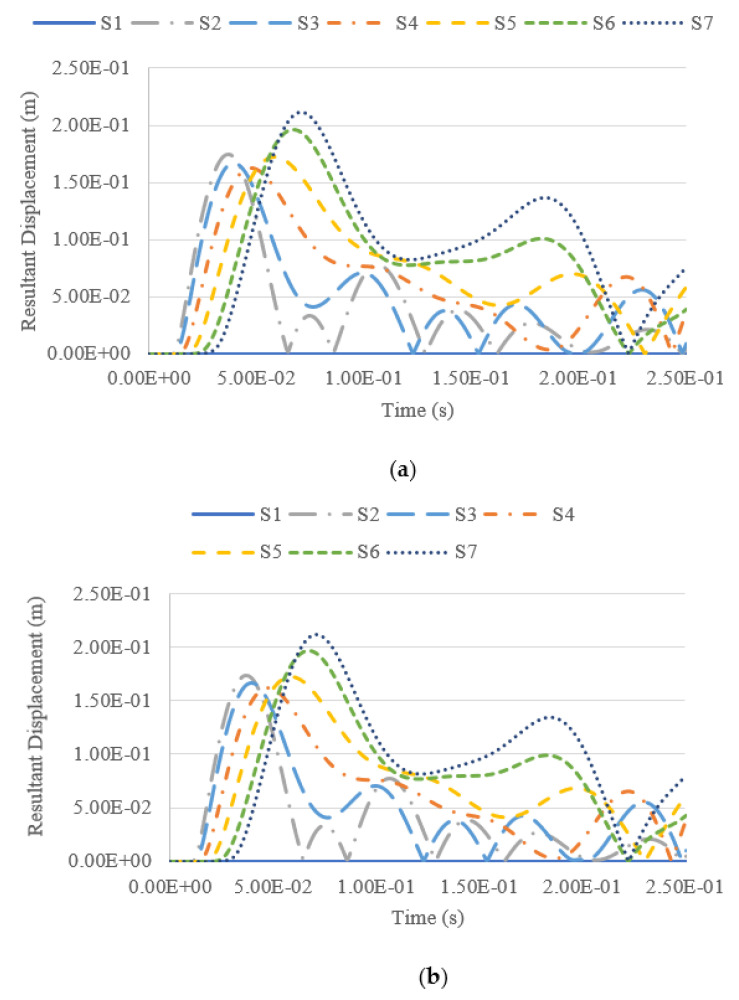
Resultant displacement versus time under blast intensity of 380 kg TNT equivalent mass 12.5 m away, (**a**) 100% fill and (**b**) 0% fill.

**Figure 17:**
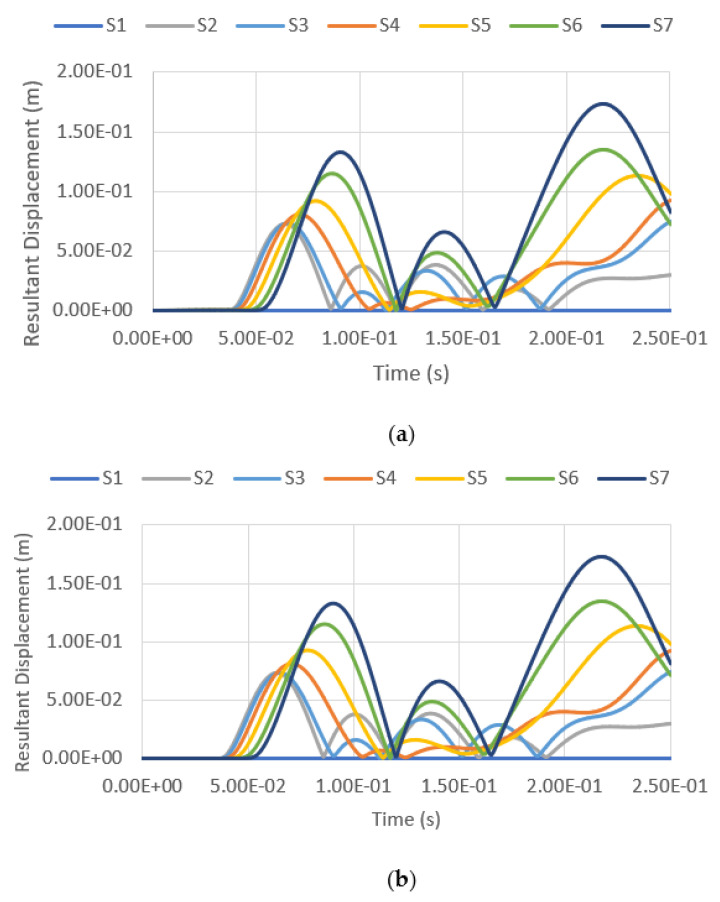
Resultant displacement versus time under blast intensity of 380 kg TNT equivalent mass 25 m away, (**a**) 100% fill and (**b**) 0% fill.

The resultant displacement versus time functions under blast intensities for different TNT equivalent masses at various fill levels and from various distances are presented in Figure 14, Figure 15, Figure 16 and Figure 17. Generally, one can observe that the resultant deformation for the different loading cases started with zero (as the blast wave had not reached the structure), and then it increased and fluctuated when the blast wave hit the structure. In general, the location of maximum displacement was always found to be at S7, which is at the top of the tank, as it is the furthest point from the support, which is at the base of the tank. Similar observations were also reported in [3]. The only exceptional case was when the blast intensity was 1500 kg TNT equivalent with a standoff distance of 12.5 m (Figure 14), in which the highest deformation was experienced at S3, located at a 7.5-m height, approximately at one-third of the total tank height. Generally, it was found that at higher-scaled distances, the maximum resultant displacement occurred at the top of the tank wall, while the lower part of the tank was subjected to greater displacement mostly in the case of low-scaled distances. The blast intensity and standoff distance have a great influence on the storage tank’s displacement, as presented in Figure 14, Figure 15, Figure 16 and Figure 17. The displacement of the tank wall increases at a lower standoff distance of the same blast intensity as compared to a greater distance. In addition, the results show that under the same standoff distance, the tank wall will deform with greater intensity if a higher blast intensity is applied. Further, the simulation results shows that the fill level has comparatively negligible effects on the overall deformation because the hydrocarbon product contained by the tank has a density of 800 kg/m^3^, which is relatively low.

### 4.5. Tank Equivalent Stress under Blast Impacts

**Figure 18:**
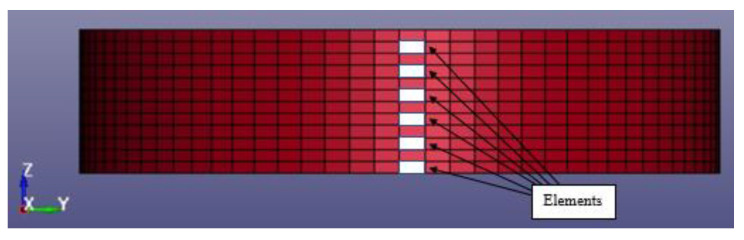
Location of the elements measured for equivalent stress.

This section discusses equivalent stress under blast loading. The equivalent stress of the tank was measured and compared with the yielding stress of the tank wall for S450 steel, with a yield stress of 440 MPa. The time history of Von Mises stresses on the tank wall throughout the analysis period of 0.25 s was measured at six elements, namely E1–E6, at the tank surface facing the blast loading. The locations of the measuring elements are shown in Figure 18, while the coordinates of the elements along the Z-axis are presented in Table 6.

**Figure 19:**
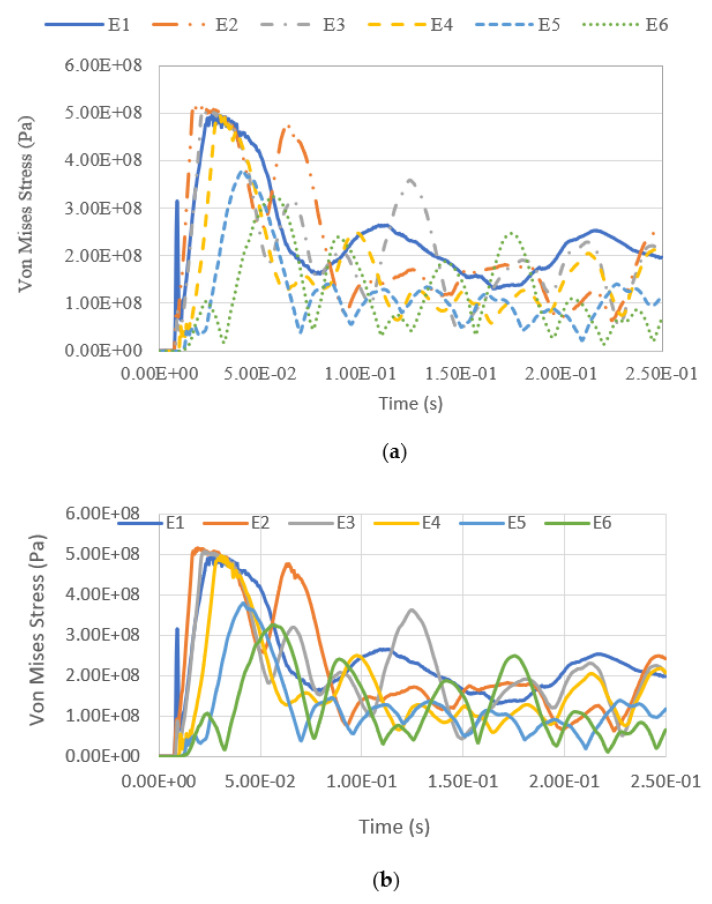
Equivalent stress versus time under blast intensity of 1500 kg TNT equivalent mass 12.5 m away, (**a**) 100% fill and (**b**) 0% fill.

**Figure 20:**
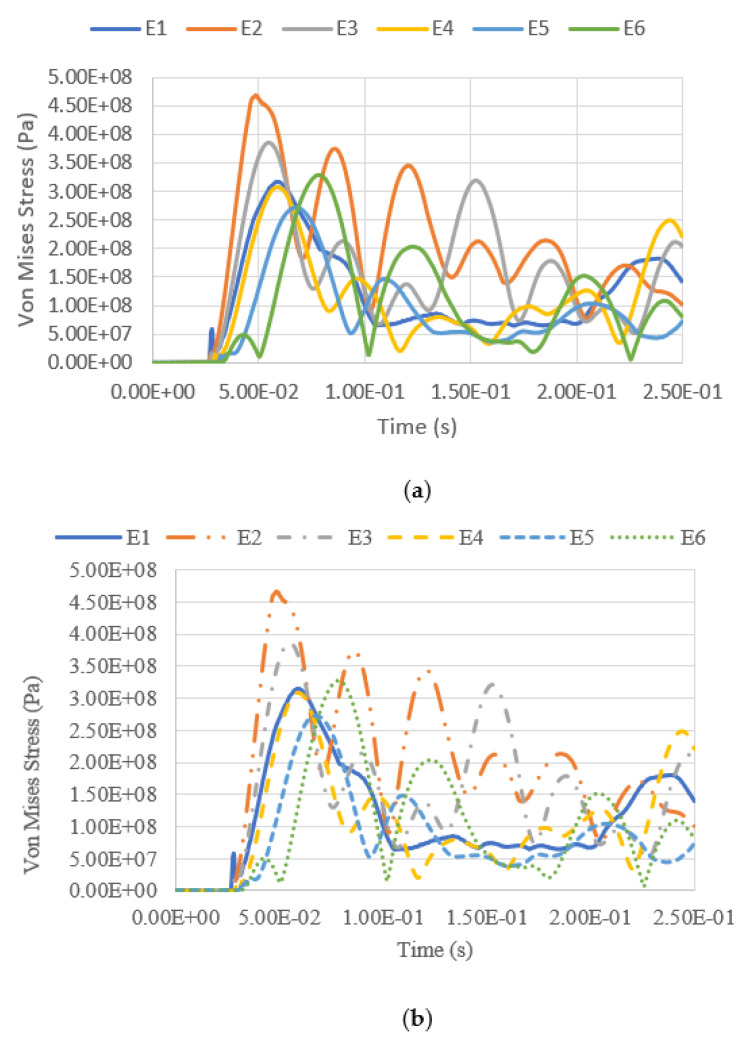
Equivalent stress versus time under blast intensity of 1500 kg TNT equivalent mass 25 m away, (**a**) 100% fill and (**b**) 0% fill.

**Figure 21:**
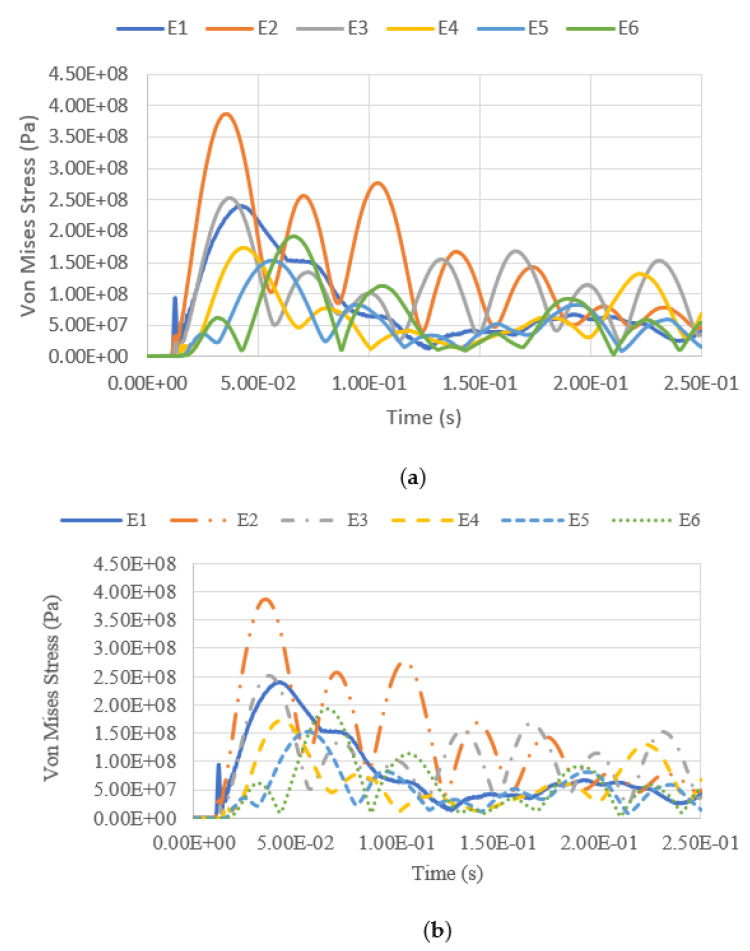
Equivalent stress versus time under blast intensity of 380 kg TNT equivalent mass 12.5 m away, (**a**) 100% fill and (**b**) 0% fill.

**Figure 22:**
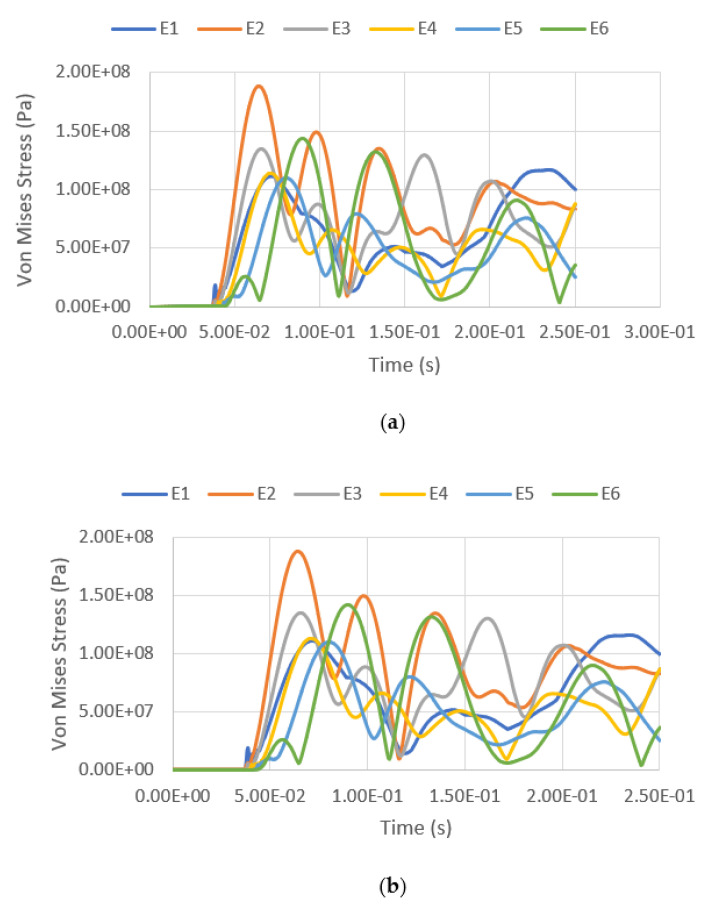
Equivalent stress versus time under blast intensity of 380 kg TNT equivalent mass 25 m away, (**a**) 100% fill and (**b**) 0% fill.

The variation of equivalent stress versus time for a blast intensity of 1500 kg TNT equivalent mass at standoff distances of 12.5 m and 25 m for different fill levels are presented in Figure 19, Figure 20, Figure 21 and Figure 22. The graphs indicate that the highest Von Mises stresses for the different loading cases are always observed at element E2, as it is closer to the charge of the blast. The first surface that will be loaded from the blast wave is the building’s front façade. This observation is in good agreement with the findings report by [28], as the first surface that will be loaded from the blast wave is the front façade of the structure. Therefore, this region should be given extra attention. It was found that the fill level has minimal influence on the Von Mises stress as well, as the pressure from the low-density hydrocarbon product contained is insignificant compared to the blast pressure. The maximum equivalent stress of each pressure was compared with the yield strength of S450, which is 440 MPa. The graphs show that the tank was unyielding under the lower blast intensity of 380 kg TNT equivalent mass at the standoff distances of 12.5 m and 25 m. However, when the tank was subjected to a blast with an intensity of 1500 kg TNT equivalent mass, the Von Mises stress exceeded the yield stress of the material. The summary of the maximum equivalent stress of the storage tank under various blast loadings is shown in Table 7.

## 5. Conclusions

This paper explored the behaviour of thin-walled cylindrical shell storage tanks subjected to blast loading using finite element methods. First, to better understand the mechanical behaviour of a thin-walled cylindrical tank, a parametric study was carried out on the storage tanks considering different material grades as well as various wall thickness and fill levels. The thicknesses of the shell elements were defined as 10, 20, 30, and 40 mm, respectively, based on the specifications in API 650. The inner face of the wall was subjected to hydrostatic pressure of 25, 50, 75, and 100% fill level based on a fluid density of 800 kg/m^3^ and kinetic viscosity of 1.9 mm^2^/s. Next, the parametric study was validated using simplified empirical equations of the thin-walled cylindrical storage tank. The numerical simulation was conducted using LS-DYNA, where the structural behaviours of the storage tank, such as blast pressure, deformed shape, resultant displacement, and equivalent stress, were obtained. The outcomes of the results show that the deformation of the tank increases with higher blast intensity, and it reduces with a greater blast standoff distance. Generally, the maximum displacement of the tank occurs at the top of the tank. The results show that Von Mises stress on the tank increased at higher blast intensity and lower standoff distance. The study also suggests that the thin-walled storage tank will yield under the blast condition of a 1500-kg TNT equivalent mass explosion at standoff distances of 12.5 m and 25 m. The results also show that the fill level has comparatively minimal influence on the displacement and the equivalent stresses of the tank, as the fluid contained has a comparatively low density. The material grade of S450 with a wall thickness of 20 mm was found to be the most efficient under the blast loads effects.

## Figures and Tables

**Table 1:** Design parameters for the thin-walled cylindrical storage tank.

**Tank Height**	22.5 m			
**Outer Diameter**	100 m			
**Wall Thickness**	10 mm	20 mm	30 mm	40 mm
**Material Yield Strength, *fy***	235 MPa	275 MPa	355 MPa	440 MPa
**Young’s Modulus, *E***	206 GPa			
**Density,** ***ρ***	7850 kg/m^3^			
**Poisson Ratio**	0.28			
**Constraints**	Fixed bottom plate and free at top			

**Table 2:** Scaled distance and arrival time of the analysis carried out.

Blast Intensity of TNT Equivalent (kg)	Standoff Distance (m)	Scaled Distance (m/kg^1/3^)	Arrival Time (s)
380	12.5	1.7258	0.0115
380	25	3.4515	0.0374
1500	12.5	1.0920	0.0079
1500	25	2.1840	0.0270

**Table 3:** Comparison between blast pressures from numerical and analytical approaches.

Blast Intensity of TNT Equivalent (kg)	Standoff Distance (m)	Peak Pressure from Numerical Modelling, (N/m^2^)	Peak Pressure from Brode Equation, (N/m^2^)	Percentage Difference with Numerical Modelling
380	12.5	240,102	217,268	9.5%
380	25	57,695	52,789	8.5%
1500	12.5	623,599	658,689	5.6%
1500	25	147,095	129,409	12%

**Table 4:** Summary of maximum tank displacement at 0.25 s.

Blast Conditions	Standoff Distance (m)	Fill Level (%)	Maximum Displacement (mm)
1500 kg TNT Equivalent	12.5	100	443.5
1500 kg TNT Equivalent	12.5	0	439.7
1500 kg TNT Equivalent	25	100	195.8
1500 kg TNT Equivalent	25	0	194.7
380 kg TNT Equivalent	12.5	100	105.0
380 kg TNT Equivalent	12.5	0	104.4
380 kg TNT Equivalent	25	100	106.4
380 kg TNT Equivalent	25	0	105.6

**Table 5:** Z-coordinates of measuring points.

Measuring Points	Z-Coordinate (m)
S1	0
S2	3.75
S3	7.5
S4	11.25
S5	15
S6	18.75
S7	22.5

**Table 6:** Z-coordinates of the elements.

Elements	Z-Coordinate (m)
E1	0–1.875
E2	3.75–5.625
E3	7.5–9.375
E4	11.25–13.125
E5	15–16.875
E6	18.75–20.625

**Table 7:** Table summary of maximum equivalent stress of the storage tank under blast loadings.

Blast Intensity of TNT Equivalent (kg)	Standoff Distance (m)	Fill Level (%)	Maximum Equivalent Stress (MPa)	Location of Maximum Equivalent Stress	Time (s)
1500	12.5	100	516.57	E2	0.0189
1500	12.5	0	516.56	E2	0.0189
1500	25	100	467.27	E2	0.0484
1500	25	0	467.15	E2	0.0484
380	12.5	100	387.21	E2	0.0354
380	12.5	0	386.16	E2	0.0354
380	25	100	188.47	E2	0.0639
380	25	0	188.49	E2	0.0639

## Data Availability

Not applicable.
